# Model-free classification of X-ray scattering signals applied to image segmentation

**DOI:** 10.1107/S1600576718011032

**Published:** 2018-09-10

**Authors:** V. Lutz-Bueno, C. Arboleda, L. Leu, M. J. Blunt, A. Busch, A. Georgiadis, P. Bertier, J. Schmatz, Z. Varga, P. Villanueva-Perez, Z. Wang, M. Lebugle, C. David, M. Stampanoni, A. Diaz, M. Guizar-Sicairos, A. Menzel

**Affiliations:** a Paul Scherrer Institut, 5232 Villigen PSI, Switzerland; b ETH Zurich, 8092 Zurich, Switzerland; c Department of Earth Science and Engineering, Imperial College London, London SW7 2BP, UK; d Shell Global Solutions International B.V., 2288 GS, Rijswijk, The Netherlands; e Lyell Centre for Marine and Earth Science and Technology, Heriot-Watt University, Edinburgh EH14 4AP, UK; f Department of Chemical Engineering, Imperial College London, London SW7 2BP, UK; g Clay and Interface Mineralogy, RWTH Aachen, 52062 Aachen, Germany; h Microstructure and Pores GmbH, 52064 Aachen, Germany; i Institute of Pathology and Molecular Pathology, University Hospital Zurich, 8092 Zurich, Switzerland; j Deutsches Elektronen-Synchrotron, Center for Free-Electron Laser Science, 22607 Hamburg, Germany

**Keywords:** polarized resonant soft X-ray scattering, anisotropic nanostructures, electromagnetic modeling

## Abstract

In most cases, the analysis of small-angle and wide-angle X-ray scattering (SAXS and WAXS, respectively) requires a theoretical model to describe the sample’s scattering, complicating the interpretation of the scattering resulting from complex heterogeneous samples. This is the reason why, in general, the analysis of a large number of scattering patterns, such as are generated by time-resolved and scanning methods, remains challenging. Here, a model-free classification method to separate SAXS/WAXS signals on the basis of their inflection points is introduced and demonstrated. This article focuses on the segmentation of scanning SAXS/WAXS maps for which each pixel corresponds to an azimuthally integrated scattering curve. In such a way, the sample composition distribution can be segmented through signal classification without applying a model or previous sample knowledge. Dimensionality reduction and clustering algorithms are employed to classify SAXS/WAXS signals according to their similarity. The number of clusters, *i.e.* the main sample regions detected by SAXS/WAXS signal similarity, is automatically estimated. From each cluster, a main representative SAXS/WAXS signal is extracted to uncover the spatial distribution of the mixtures of phases that form the sample. As examples of applications, a mudrock sample and two breast tissue lesions are segmented.

## Abstract 2

This article describes a modeling framework to relate the molecular orientation of nanostructures to polarized resonant soft X-ray scattering measurements using the Born approximation and a full tensor treatment.

## 1. Introduction   

The high flux of modern light sources allows small-angle X-ray scattering (SAXS) and wide-angle X-ray scattering (WAXS) measurements to proceed rapidly and to produce significant data sets, often with a continuous sampling rate in excess of 10 Hz. From each such measurement, parameters characteristic of the sample, such as composition, homogeneity, and particle size and shape, can be extracted. The interpretation of such high data volumes generated by scanning and time-resolved SAXS/WAXS methods is facilitated by statistical approaches. There are multiple data analysis routines for extracting information from scattering curves, defined as the scattered photon intensity 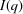 as a function of the magnitude of the scattering vector **q**, based on models from scattering theory and statistical mechanics [*q* = |**q**| = (4π/λ)sinθ, where θ is half the scattering angle and λ is the wavelength of the incident radiation]. The decoupling approach is one example that considers geometrical models of form factor 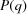 and structure factor 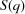 to perform a least-squares fit to 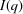. However, this method is essentially only applicable to dilute, monodisperse and homogeneous samples. As a consequence, the scattering curves 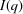 of multiphase, heterogeneous, bulk or solid samples cannot be easily modeled using standard data analysis routines even though they are common samples for SAXS/WAXS. The modeling of multiphase samples is extremely complex when each independent phase of a sample is unknown and inseparable from the bulk. Common approaches for assessing mixtures require prior knowledge of the independent components, simple models or some symmetry relationship (Kozielski *et al.*, 2001 ▸; Konarev *et al.*, 2003 ▸; Petoukhov *et al.*, 2012 ▸; Breßler *et al.*, 2015 ▸).

Life and materials science samples are usually composed of numerous phases, which can have different structural conformations and chemical compositions. When an X-ray beam passes through such a sample, all phases along the beam path will cumulatively contribute to the overall scattering pattern. Advances in the analysis of structural conformation and chemical composition of SAXS/WAXS signals of heterogeneous samples are fundamental to simplify the interpretation of large SAXS/WAXS data sets. Molecular anisotropy is a common qualitative structural feature extracted from scanning SAXS/WAXS that can be recovered over extended sample regions and described without recourse to other sample properties. This approach provides information about the sample’s structure and is valid even for mixed phases if anisotropic scattering features of independent phases are present in different *q* ranges (Bunk *et al.*, 2009 ▸). Large data sets are frequently reduced by singular value decomposition (SVD) (Hansen, 1987 ▸), which has been applied to SAXS to determine the number of distinct scattering species and their relative abundance (Henry & Hofrichter, 1992 ▸; Segel *et al.*, 1998 ▸). However, SVD does not easily lend itself to constructing the scattering profile of these components. An alternative approach is canonical correlation analysis, which is a statistical method to quantify the degree of linear dependence between two variables. Applying it to scattering curves 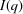 of measured mixed phases and the recovered independent signals from a SAXS/WAXS data set (Guagliardi *et al.*, 2007 ▸), one can obtain information on the composition of the sample.

There are two main acquisition modes that produce large SAXS/WAXS data sets: (i) time resolved, when monitoring particle size, abundance and shape variations as a function of time, as in the case of crystal nucleation and nanoparticle formation; and (ii) spatially resolved, when mapping variations in composition of a solid sample, *e.g.* body tissues and composites. Our motivation is to identify, monitor and classify the temporal and spatial variations of SAXS/WAXS signals independently of geometrical models and assumptions.

The versatile statistical method presented here can be applied to different samples and experimental data sets. We apply it to the segmentation of spatially resolved scanning SAXS/WAXS measurements to segment automatically distinct sample regions on the basis of the similarity of the SAXS/WAXS signal. A focused X-ray beam offers high spatial resolution, which is of particular interest for heterogeneous samples. The segmentation of two different sample types is demonstrated: (i) an inorganic mudrock slice and (ii) breast tissue lesions in the presence of microcalcifications.

High-resolution imaging, such as transmission and scanning microscopy, often focuses on small areas of thin samples to reach nanoscale resolution. For large heterogeneous samples scanning SAXS/WAXS has the advantage that it probes the sample’s nanostructure over relatively large areas of mm^2^ with minimum sample preparation, as no dehydration, staining or embedding of the sample is required (von Gundlach *et al.*, 2016 ▸; Sibillano *et al.*, 2016 ▸). Scanning a sample with an X-ray beam leads to rich structural and compositional information. To facilitate interpretation of scanning SAXS measurements, parameter maps can be derived from the integrated scattering curves. These parameter maps will represent the spatial distribution of a structural or compositional quantity of a heterogeneous sample and can be represented as pixels of an image.

Multiphase signals combined with the large data sets of scanning and time-resolved SAXS/WAXS complicate the extraction of quantitative composition-dependent information (Altamura *et al.*, 2016 ▸). If independent signals from pure phases can be measured, the linear combination can be calculated to represent the SAXS/WAXS signal (Ladisa *et al.*, 2007 ▸). However, our focus lies in the scenario where signals of pure phases are difficult to find, which is a common problem in biology and materials science applications. The identification of how many phases and in what ratio they form a sample requires the decoupling of the measured SAXS/WAXS intensity into independent signals.

## 2. Experiments and methods   

### 2.1. Sample preparation   

#### 2.1.1. Mudrock sample   

A section of approximately 25 µm thickness was prepared from an outcrop mudrock sample from the Eagle Ford formation by Microstructure and Pores GmbH in Aachen, Germany. The rock slice was mounted with its layering perpendicular to the surface of a silicon wafer. The sample surface was polished using a standard thin-section preparation protocol under dry atmospheric conditions, followed by argon broad ion beam polishing with a Leica EM TIC 3X triple ion beam cutter. Polishing was undertaken at low energy and in short intervals of 30 min, alternating with breaks of minimum 60 min to prevent intense heating of the sample.

#### 2.1.2. Breast tissue sample   

Two human breast tissue samples from the same patient were provided by the Institute of Pathology and Molecular Pathology of the University Hospital Zurich, Switzerland. Informed consent was obtained from the donor. The samples were fixed in 4% buffered formalin and examined through radiographic measurements on an X-ray tube. Slices of 1–2 mm were cut in the regions where microcalcifications were observed. The slices were fixed on a sample holder using Kapton foil and tape.

### 2.2. Scanning SAXS/WAXS   

**Figure 1:**
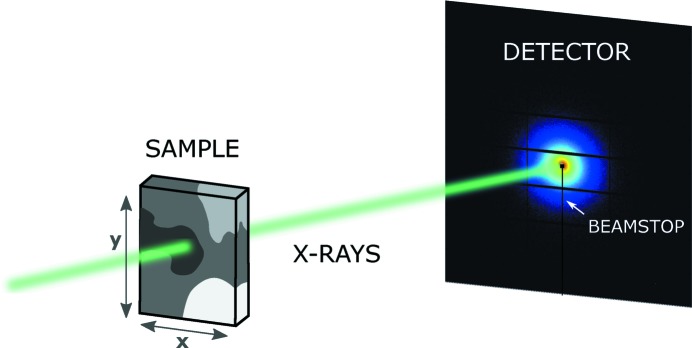
Experimental setup: The sample is scanned across an X-ray beam along **x** and **y**. Scattering patterns are measured by a two-dimensional detector at each scanning point. The beamstop protects the detector from the direct X-ray beam.

Scanning SAXS/WAXS measurements of both samples were carried out at the cSAXS beamline of the Swiss Light Source (SLS) at the Paul Scherrer Institut in Villigen, Switzerland. To accelerate the acquisition, a continuous scan mode was employed, in which the sample moves at constant speed along the **y** direction while the detector records data continuously (see Fig. 1 ▸). After the line is finished, the sample moves by a step in **x** and the continuous scan restarts along **y**. The mudrock sample was scanned at an energy of 11.48 keV, using a beam of *x* = 5 µm and *y* = 2 µm focused by elliptical Fresnel zone plates (Lebugle *et al.*, 2017 ▸) with an approximate flux in the sample position of 6.22 × 10^9^ photons s^−1^. The scan step or image pixel size was 5 × 5 µm. The sample-to-detector distance was 0.32 m. We refer to the covered *q* range of *q* = 5–32 nm^−1^ as WAXS, which allows us to measure the Bragg peaks of the minerals. A Pilatus 2M detector (Henrich *et al.*, 2009 ▸) was used to acquire scattering patterns at a rate of 3.4 Hz, with an exposure time of 290 ms and detector readout of 5 ms per frame. The two-dimensional scattering patterns were azimuthally integrated and their intensity normalized by the sample transmission relative to air. The scattering of the silicon wafer used as sample holder was subtracted as background. The breast tissue samples were scanned at an energy of 11.20 keV with an approximate flux of 1.27 × 10^11^ photons s^−1^ in the sample position, using a beam of *x* = 30 µm and *y* = 20 µm, which was focused by bending the second monochromator crystal and the high-order-rejection vertical mirror of the beamline. The scan step and pixel size were set to 30 × 30 µm. The sample-to-detector distance was 2.16 m. We refer to the covered *q* range of *q* = 0.07–4 nm^−1^ as SAXS. The scattering patterns were acquired at a rate of 25 Hz, with an exposure time of 35 ms and a detector readout time of 5 ms per frame.

### 2.3. Energy-dispersive X-ray spectroscopy   

For the mudrock sample, energy-dispersive X-ray spectroscopy (EDX) was performed with an Xmax150 EDX detector from Oxford Instruments at an acceleration voltage of 15 kV.

### 2.4. X-ray diffraction   

Powder X-ray diffraction (XRD) was carried out on the same mudrock sample by the Clay and Interface Mineralogy group at Aachen University, Germany, following the experimental protocol reported by Seemann *et al.* (2017 ▸).

## 3. Results and discussion   

### 3.1. Mudrock sample: data analysis procedure   

**Figure 2:**
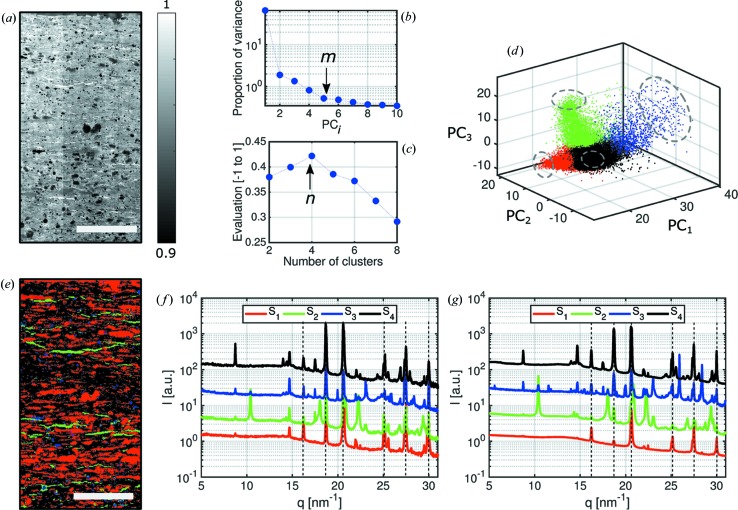
(*a*) X-ray transmission map of a thin mudrock slice. The scale bar represents 1 mm. (*b*) L-curve resulting from principal component analysis. It displays the proportion of variance explained as a function of principal component PC*_j_*, for 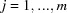, where *m* is the number of considered principal components. (*c*) Evaluation of the optimal number of clusters. From the silhouette criterion, the data set is best classified into four clusters. (*d*) Classification of WAXS signals into four clusters. The clusters are not isolated. Thus, transition regions prone to misclassification are observed. (*e*) Segmentation of scanning WAXS data according to clustering results. (*f*) Representative signals extracted as the nearest point to the cluster’s centroid. For readability, the signals are shifted along the *y* axis. (*g*) Representative signals extracted as an average of the furthest points from the centroids in each cluster. These regions are represented by the dashed circles in (*d*). For readability, the signals are shifted along the *y* axis.

**Figure 3:**
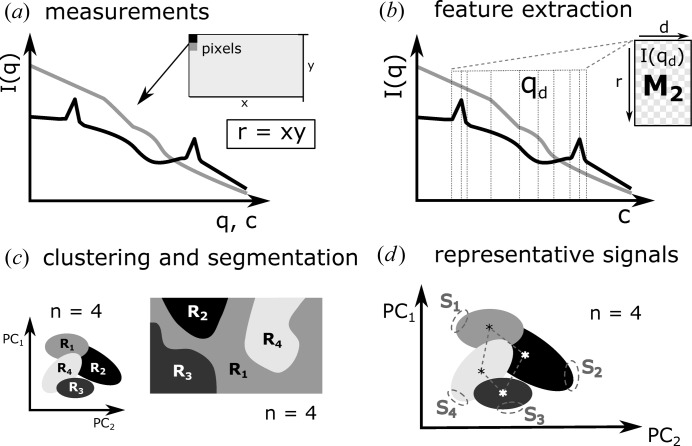
Scheme of the data analysis procedure. (*a*) Collection of azimuthally integrated SAXS/WAXS intensity curves 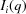, for 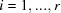, where *r* is the number of 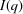 points. (*b*) **M**_2_ is formed by selecting only the *d* intensities 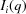 where inflection points occur. (*c*) Dimensionality reduction: principal component analysis is applied to **M**_2_ and the number of main variables is reduced to *m* principal components PC*_j_*, for 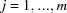. The optimal number of clusters *n* is evaluated and the signals are classified into *n* clusters. We assume *n* = 4 in this example. The scanned map is segmented according to the clustering results. (*d*) Estimation of a representative signal 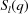 by selecting points that are located furthest from all centroids, for 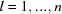. Further details about each step can be found in the text.

To demonstrate our algorithm, we employ scanning WAXS measurements of a mudrock slice (Fig. 2 ▸). As a reference for the spatial distribution of intensities, the transmission map of the scanned region is shown in Fig. 2 ▸(*a*). The 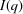 curves are sensitive to the sample’s structural and crystalline composition, and the resulting WAXS mapping provides the two-dimensional spatial phase distribution of the sample. Here we aim to automatically classify the large data sets of mixed-phase signals 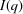 obtained from SAXS/WAXS measurements. One possible approach is based on the similarity of the scattering curves. Fig. 3 ▸ represents the main steps of the data analysis procedure presented here. All analysis steps described herein were developed and tested using in-house-developed routines of cSAXS in MathWorks MATLAB v2016b. The codes can be found on the cSAXS web page at https://www.psi.ch/sls/csaxs/software. The input data were prepared from measured SAXS/WAXS signals (Fig. 3 ▸ *a*) as an *r* × *q* matrix 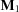, where *r* is the number of 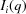 curves and *q* is the range of scattering vector moduli in 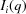. To avoid weighting and misclassifying 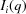 signals on the basis of magnitude, indicative of variations of sample thickness for instance, each scattering curve was normalized by its intensity averaged over the measured *q* range. The goal of such a normalization is to bring all scattering curves to the same order of magnitude, as our method focuses mostly on the scattering curve’s shapes. For other scanning SAXS/WAXS analysis the common normalization employing the measured transmission map may be recommended. To decrease the high-frequency noise, 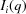 was resampled by logarithmically reducing the total number of *q* values by a factor of ten, leading to a total of *c* values.

Fig. 3 ▸(*b*) shows a scheme of the feature extraction step. We focus only on the scattering intensities at the inflection points, where the first or second derivatives are zero, *i.e.* 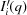 = 0 or 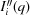 = 0. We select those intensities as the interesting structural information in scattering curves is often characterized by Bragg peaks and slope variations. Extracting only such inflection points, represented by *q*_d_ in Fig. 3 ▸(*b*), provides succinct structural information about the geometric shape of the scattering signals (Zamani & Demosthenous, 2014 ▸). This simplifies the classification of scattering curves, as the data set is reduced to the important features 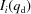, instead of using all available 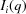 values. Feature extraction reduces the input matrix to 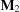 with dimensions of *r* × *d*, where *d* is the number of features with 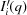 = 0 or 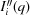 = 0 and *d* < *c* (Fig. 3 ▸ *b*). Some intensity curves will have no inflection or peaks in the selected *q*_d_ ranges. The columns of *d* related to such signals will be completed with zeros.

Principal component analysis (PCA) is applied to further reduce the number of variables *d* (Hansen, 1987 ▸). PCA extracts the linearly uncorrelated signals in 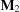, reducing the data set to *m* variables that contain the most significant signals in order of decreasing representativeness. These are known as the principal component coefficients PC*_j_*, for 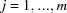, where *m* is the number of considered principal components. The first principal component, PC_1_, accounts for the function that generates the maximum variance of the data set when the component is removed. The second principal component, PC_2_, accounts for as much of the remaining variance as possible, with the constraint that the correlation between PC_1_ and PC_2_ is zero. Any further component will maximize the variance of the residual data while being uncorrelated from all the lower-order components. The number of principal component coefficients, *m*, is obtained from an L-curve by plotting the proportion of total variance accounted for by PC*_j_* as a function of the number of principal components. Fig. 2 ▸(*b*) plots the L-curve of the first ten principal components resulting from PCA analysis of the mudrock sample. Here, the number of components to represent the data set, *m*, is defined by the knee position on the horizontal axis. Using the L-curve method, *m* = 5 components account for more than 95% of the variance of the data set.

The selected principal component coefficients are then classified in clusters by *k*-means (Lloyd, 1982 ▸). To evaluate the optimal number of clusters, *n*, we apply the silhouette criterion (Rousseeuw, 1987 ▸). Using a Euclidean distance metric, each signal is assigned a silhouette value within the range between −1 and 1. A high value indicates a good match of the signal to its own cluster compared to the distance to other clusters. We calculate the average over all points’ silhouette values for a range of numbers of clusters (Fig. 2 ▸ *c*). In our experience, it has been sufficient to select the number of clusters *n* which maximizes this value without the need of intervention or prior knowledge or assumptions. For our test sample, we tested the range of 2–8 clusters and concluded that the WAXS signals are best classified in four clusters, representing four main components of the sample, as schematically shown in Fig. 3 ▸(*c*).

Each cluster resulting from *k*-means represents a main type of signal that is present in the data set. If clusters are completely isolated, they represent independent phases of a sample. In the simplified two-dimensional representation of *k*-means clustering in Fig. 3 ▸(*c*), only the principal components PC*_j_* with 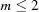 are represented. If clusters are not isolated and have an interface, a gradual transition occurs between the representative signals from one to another, indicating that the signals are mixed at the interface and cannot be separated. For visualization purposes, the *k*-means clustering is shown in three dimensions for our test sample in Fig. 2 ▸(*d*) with only the first three of five principal components plotted. This graphical representation shows non-isolated clusters, and in this case, only the main mixtures of phases, which follow different trends, can be classified.

The classification of each scattering curve within a certain cluster is used to divide the scanning SAXS/WAXS measurements into *n* segments, as sketched in Fig. 3 ▸(*c*). Each pixel of the scanned region will correspond to a cluster. Following this, the scanned map can be segmented according to regions with similar scattering signals. For the test sample, the segmentation according to the classification of signals in four clusters is shown in Fig. 2 ▸(*e*). The colors correspond to the clusters previously defined in Fig. 2 ▸(*d*).

We follow two main automated approaches to find a representative signal 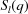 for each cluster. The first considers the cluster’s centroid, which is defined as the cluster’s ‘core’, representing the average between all the signals classified within it. The centroids are schematically represented in Fig. 3 ▸(*d*) by asterisks. Fig. 2 ▸(*f*) shows the four scattering curves from the test sample related to 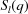, identified as the closest point to the cluster’s centroids (Fig. 2 ▸ *d*). If a cluster encloses signals from mixed phases, its most uncorrelated signals will have the highest probability of representing signals from pure phases. This leads to the second approach, in which we identify 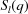 as a set of points located the furthest from the centroids of all clusters, as represented in Fig. 3 ▸(*d*). Considering the largest distances between points in a certain cluster and the centroids of all clusters, we select the most uncorrelated signals, thereby avoiding the overlapping interfaces between clusters, which are prone to misclassification. We select 10% of all the points within a cluster which have the largest Euclidean distances from the centroids. As a visual aid, we indicate the points around the cluster’s outer limits within the dashed circles in Fig. 2 ▸(*d*), which were selected as the representative signals of the mudrock sample, shown in Fig. 2 ▸(*g*). If a cluster is related to a homogeneous region of the sample, *i.e.* formed by a pure phase, and if this pure-phase region is large enough to generate sufficient statistics to be classified into an independent and isolated cluster, its representative signal 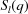 will represent a pure phase. However, often the illuminated volume of a sample is composed by mixed phases, and thus 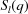 will only represent scattering signals of mixtures that form such a heterogeneous sample.

As expected from a geological sample measured by scanning WAXS, no pure phases were obtained from the segmentation of our test sample (Leu *et al.*, 2016 ▸). The Bragg peaks in Fig. 2 ▸(*g*) can only be explained by mixtures of mineral phases. However, characteristic mixtures in the sample as well as their spatial distribution can be determined. All representative signals exhibit a set of common peaks, resulting from a mixture of quartz and calcite phases. They are indicated by the vertical dashed lines in Figs. 2 ▸(*f*) and 2 ▸(*g*). The choice of representative signals, which were selected as the furthest signals from the cluster centroid’s, is confirmed by XRD which provides the sample’s composition with higher resolution and better signal-to-noise ratio compared to scanning WAXS. The sample composition is estimated from Rietveld refinement on the basis of the crystalline structure. The main phases determined by XRD are 58.8% of calcite, 20% of quartz, 5.3% of kaolinite, 5.3% of pyrite, 3% of illite/smectite, 3.3% of gypsum and a total organic carbon content of 4.3%. For further discussion of the representative signals, we focus only on the peaks that distinguish the mixtures in Fig. 2 ▸(*g*).

**Figure 4:**
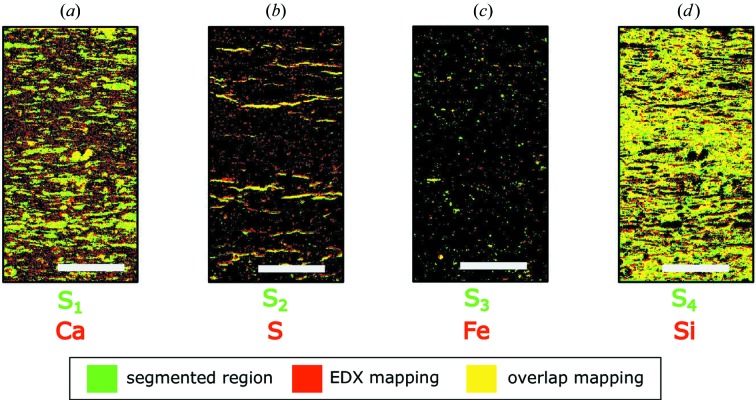
Results of WAXS segmentation for the mudrock sample. WAXS image segmentation is represented in green, while red corresponds to superficial EDX mappings. Areas where these maps overlap are represented in yellow. (*a*) Segmentation of representative signal *S*_1_ and EDX mapping of calcium. (*b*) *S*_2_ and sulfur. (*c*) *S*_3_ and iron. (*d*) *S*_4_ and silicon.

In Figs. 4 ▸(*a*)–4 ▸(*d*), we compare the results of WAXS segmentation, in green, with the element-sensitive EDX mapping of the same region, in red. Yellow regions indicate where the segmentation and EDX mappings overlap. The Bragg peaks in the representative signal *S*_1_ in Fig. 2 ▸(*g*) correspond well to the diffraction pattern of calcite, which makes up 58.8% of the mudrock sample. This exemplifies that a signal could be independently segmented if its phase generates enough statistics. The overlapping region between the *S*_1_ segment and the EDX mapping of calcium in Fig. 4 ▸(*a*) confirms that the segmented region correlates to the spatial distribution of calcite. Differences between EDX and segmentation mappings stem from the distinct volumes probed by the two techniques. EDX probes the surface of the mudrock sample, whereas WAXS signals are an average of the composition over the whole depth of the illuminated sample volume.

The representative signal *S*_2_ leads to the segmented image in Fig. 4 ▸(*b*), which correlates to the EDX surface mapping of sulfur. According to powder XRD, 3.3% of the sample composition is gypsum, although the WAXS peaks indicate a phase transition to bassanite, probably caused by ion-beam polishing during sample preparation or radiation damage during X-ray exposure. Even though the representative signal *S*_3_ corresponds to a mixture, it exhibits the main peaks of pyrite, which makes up 5.3% of the sample. Its segmentation in Fig. 4 ▸(*c*) correlates to surface regions rich in iron. The representative signal *S*_4_ includes the remaining mixtures, but its features have no unique correspondence to any of the components. The clustering in Fig. 2 ▸(*d*) confirms that the region *S*_4_ shares its interfaces with all other clusters. The segmentation in Fig. 4 ▸(*d*) correlates to the EDX mapping of silicon, owing to the presence of quartz and clay minerals. The comparison between WAXS, EDX and XRD confirms that the mixture of phases that compose the mudrock sample could be segmented. The overlap of surface EDX and segmented WAXS mappings confirms the significance of the segmented sample regions. We show that finding the main representative WAXS signals simplifies the qualitative interpretation of large data sets without the need of a model, unlike Rietveld refinement, and uncovers the spatially resolved composition distribution of complex heterogeneous crystalline samples.

Such a WAXS-based segmentation can be used to identify and label micro-domains in mudrocks, as well as their anisotropy, if specific segments of the detector are chosen. These domains can be characterized in even more detail by scanning SAXS to quantify pore orientation, preferential alignment, porosity and pore size distributions (Leu *et al.*, 2016 ▸). The method introduced here is equally applicable to the spatial mapping and identification of mineral phases and their orientation using powder diffraction or wide-angle X-ray scattering (Wenk *et al.*, 2008 ▸; Kanitpanyacharoen *et al.*, 2012 ▸; Leu *et al.*, 2016 ▸). Thus, systematic relationships between mineralogy and pore structure, and their spatial variation, can be investigated. These are required for accurate numerical modeling and prediction of fluid flow through a pore network. Several large-scale industrial applications, such as shale gas production (Gensterblum *et al.*, 2015 ▸), CO_2_ sequestration (Rutter *et al.*, 2017 ▸) and nuclear water storage (Marschall *et al.*, 2005 ▸), are limited by fluid flow in mudrocks. The segmentation will provide insight also into other heterogeneous rocks containing nanoscopic pores, such as carbon­ates, tight sandstones and coal.

### 3.2. Breast tissue lesions   

**Figure 5:**
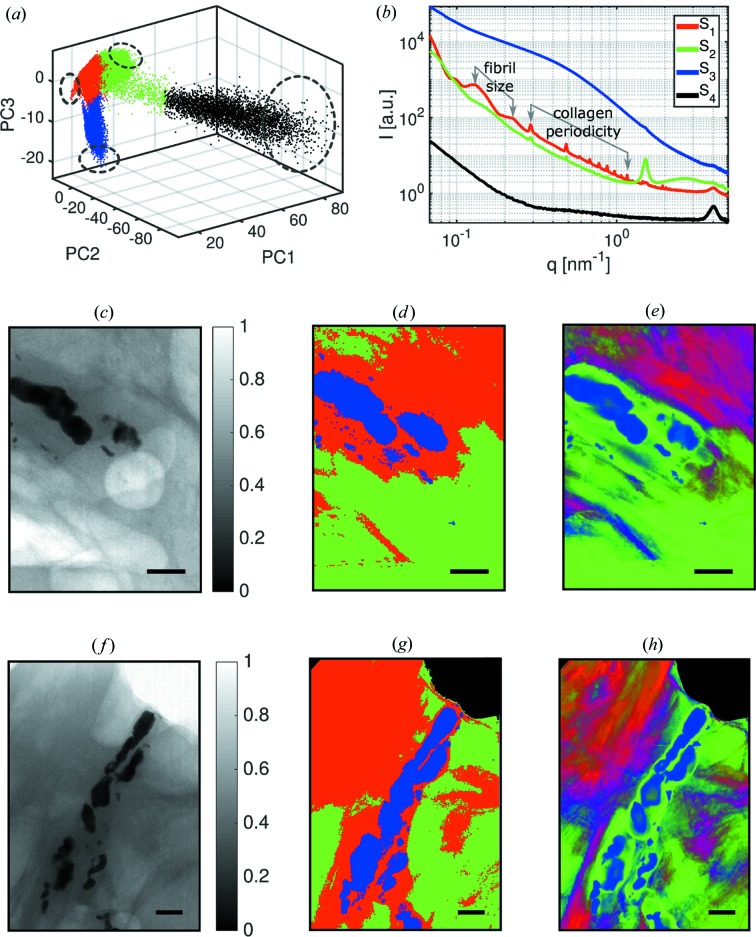
Results of scanning SAXS measurements of breast lesions. (*a*) The data set is classified into four clusters. (*b*) The representative signals for each cluster indicate that the breast tissue is segmented into regions that are rich in collagen (*S*_1_), lipids (*S*_2_), microcalcifications (*S*_3_) and Kapton (*S*_4_). *S*_4_ is recovered as a region of pure Kapton at the sample. Benign breast lesion: (*c*) Transmission map of the benign sample. (*d*) Image segmentation. (*e*) Correlation maps that indicate misclassification of signals near the interface of clusters of lipid-rich and collagen-rich regions, showing the regions of transition between clusters. Malignant breast lesion: (*f*) Transmission map of the malignant sample. (*g*) The segmentation shows a clear separation of the sample into four main regions, including the Kapton corners *S*_4_ from sample preparation. (*h*) Correlation map that emphasizes collagen-rich and lipid-rich classification of the malignant tumor.

In this section, we discuss the segmentation of scanning SAXS measurements of breast lesions containing microcalcifications (Arboleda, 2017 ▸). We apply the segmentation procedure to two samples, to increase the statistics. The clustering evaluation reached a maximum when the measured SAXS signals were classified into four clusters, labeled again *S*_1_–*S*_4_. The results of *k*-means clustering are shown in Fig. 5 ▸(*a*); the dashed circles represent the regions where the representative signals were selected. The estimated representative signals are shown in Fig. 5 ▸(*b*) for each cluster. From the known breast tissue composition (Suortti *et al.*, 2003 ▸) and with the help of the transmission map, we associate the classified signals as follows: *S*_1_ represents the scattering of collagen-rich tissues; *S*_2_ represents the scattering of lipid-rich tissues; *S*_3_ represents the scattering of microcalcifications; and *S*_4_ represents the scattering of Kapton, which served as sample support.

The representative signal *S*_1_ in Fig. 5 ▸(*b*) corresponds to collagen scattering patterns; in this figure, the scattering from the periodicity of the collagen fibrils as well as the scattering from their thickness is indicated (Suhonen *et al.*, 2005 ▸). This signal is typical of type I and III of collagen, which are abundant in breast tissue (Sidhu, 2009 ▸). Lipids are composed of triglyceride molecules packed into a hexagonal lattice. They assemble into bilayers which form lamellar structures with a spacing of about 4.26 nm (Suortti *et al.*, 2003 ▸). This corresponds to the peak found around *q* = 1.47 nm^−1^ in the representative signal *S*_2_ of Fig. 5 ▸(*b*). Microcalcifications lead to the scattering typical of hydroxyapathites, *S*_3_, which have different chemical compositions and crystalline properties (Frappart *et al.*, 1984 ▸; Radi, 1989 ▸; Haka *et al.*, 2002 ▸). Owing to sample preparation, there are Kapton regions in the top corners of Figs. 5 ▸(*f*)–5 ▸(*h*). These regions are clearly segmented by the representative signal *S*_4_, as it corresponds to a pure phase with enough statistics.

Figs. 5 ▸(*c*)–5 ▸(*e*) show the images related to a benign breast lesion and Figs. 5 ▸(*f*)–5 ▸(*h*) relate to a malignant breast lesion. According to a histo­path­ological examination, the malignant sample corresponds to a ductal carcinoma *in situ*, and the microcalcifications present in both samples were classified as calcium hydroxyapatite or type II since they showed non-birefringent properties under the microscope (Frappart *et al.*, 1984 ▸; Radi, 1989 ▸; Haka *et al.*, 2002 ▸). The transmission maps shown in Figs. 5 ▸(*c*) and 5 ▸(*f*) are visual references for the image segmentation of the microcalcifications. The segmented signals represent the main regions shown in Fig. 5 ▸(*d*) and 5 ▸(*g*).

To quantify the accuracy of the segmentation we use Pearson’s correlation coefficient ρ between two given variables *x* and *y*, calculated by 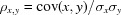, where cov is the covariance and σ is the standard deviation. Here, we calculate ρ to quantify the linear dependence between each representative signal 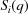 and the scattering signals 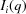 in the data set. If representative signals are linked to pure phases, the analysis can be performed by the generalized canonical correlation analysis (Guagliardi *et al.*, 2010 ▸; Giannini *et al.*, 2014 ▸; Sibillano *et al.*, 2016 ▸). Each data point 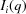 will have *n* correlation coefficients, one for each cluster. In the case of the breast tissue samples, *n* = 4. The correlation maps are a finer representation of the transmission maps in Figs. 5 ▸(*c*) and 5 ▸(*f*), especially when focusing on the shape of the microcalcifications.

The calculated correlation maps are shown in Figs. 5 ▸(*e*) and 5 ▸(*h*) for the breast tissue lesions. We show the correlation coefficients ρ of the three main phases *S*_1_, *S*_2_ and *S*_3_ with an RGB color scheme that allows for the representation of color gradients and mixtures. For graphical representation, correlation coefficients with values smaller than the median calculated for the whole image are set to zero, to avoid overlapping regions with low correlation. The segmentation of the SAXS signals is confirmed for *S*_3_ and *S*_4_ signals, comparing Fig. 5 ▸(*d*) and 5 ▸(*g*) with Fig. 5 ▸(*e*) and 5 ▸(*h*), respectively. However, there were misclassified points for regions between *S*_1_ and *S*_2_, as these transitions occur between collagen-rich and lipid-rich regions that have similar features. Lipid-rich tissue regions were misclassified especially around the microcalcifications, emphasizing the importance of calculating the correlation of signals. As previously reported (Fernandez & Keyrilainen, 2004 ▸; Fernández *et al.*, 2005 ▸), a more heterogeneous structure is detected for the malignant sample, owing to the invasion of cancer into the lipid-rich tissue.

This segmentation procedure can be used to further segment the already classified signals in Fig. 5 ▸ to find finer differences in composition and structure of benign and malignant lesions. One example could be to distinguish between microcalcification types I and II, as the type, composition and size obtained from SAXS measurements could become an indicator of the severity of breast lesions. As collagen-rich tissue can be successfully separated from lipid-rich tissue, another possibility is the determination of breast tissue density by measuring the full width at half-maximum of the lipid peak for each pixel (Sidhu *et al.*, 2011 ▸). This is an important indicator of breast cancer risk in patients (Byrne *et al.*, 1995 ▸; Boyd *et al.*, 2007 ▸, 2011 ▸). However, as discussed by Arboleda (2017 ▸), more samples from more patients are required for those applications to generate enough statistics and to avoid misclassification and misdiagnosis.

## 4. Conclusion   

We present a method to automatically classify scattering curves of SAXS/WAXS measurements according to feature extraction of their inflection points, such as the presence of Bragg peaks and slope variations. One of the main advantages of a statistical approach for SAXS/WAXS data analysis is to find similarities between signals and to classify them without the need of models, prior sample knowledge or human intervention. The optimal number of clusters, *i.e.* main sample regions, is determined by calculating the highest cluster evaluation value based on the silhouette criterion. The classification of SAXS/WAXS signals into few clusters simplifies the data set to a few representative signals that can be used for further analysis. The suitability of the method was illustrated on image segmentation of scanning SAXS/WAXS measurements of a mudrock slice and breast tissue lesion samples. The main sample regions were automatically segmented on the basis of SAXS/WAXS similarity and mapped on two-dimensional color-coded maps.

Some limitations of our method are nonlinear contributions to the scattering patterns and the nanostructural anisotropy of a sample. For example, the scattering of building blocks forming an independent phase of the sample will depend on the level of organization, composition or anisotropy; thus the same building blocks can scatter differently and even be classified as different phases. As a prospect for development, we aim to extend this data analysis procedure to higher dimensionalities and use it to segment, classify and quantify the phases present in small-angle scattering tensor tomography, applying the segmentation to three-dimensional reciprocal space (Liebi *et al.*, 2015 ▸, 2018 ▸; Schaff *et al.*, 2015 ▸).
